# Systematic optimization of PD-L1 and CLDN18.2 CAR-T designs identifies a bicistronic dual-target, double-CD3ζ architecture with enhanced antitumor activity in gastric cancer

**DOI:** 10.3389/fimmu.2026.1805471

**Published:** 2026-06-02

**Authors:** Xiang Zhang, Yao-jie Kong, Jing-yao Li, Heng-hui Li, Liang Chen

**Affiliations:** 1 School of Medicine, Shanghai University, Shanghai, China; 2 Shanghai Tenth People’s Hospital, Shanghai, China; 3 Institute of Artificial Intelligence and Biomanufacturing, School of Medicine, Shanghai University, Shanghai, China; 4 State Key Laboratory of New Targets Discovery and Drug Development for Major Diseases, Xi’an, China

**Keywords:** CAR T-cell therapy, gastric cancer, CLDN18.2, PD-L1, dual-target CAR-T, CD3ζ, bicistronic CAR, immunotherapy

## Abstract

**Background:**

Gastric cancer is a leading cause of cancer-related mortality, with limited efficacy of immunotherapies due to intratumoral heterogeneity, an immunosuppressive microenvironment, and antigen-loss escape. The optimization of chimeric antigen receptor (CAR) T-cell therapy targeting PD-L1 and Claudin18.2 (CLDN18.2) is crucial for improving the antitumor response.

**Methods:**

We performed a stepwise optimization of CAR designs targeting PD-L1 and CLDN18.2, focusing on the costimulatory domains and intracellular signaling architecture. We engineered monospecific PD-L1 and CLDN18.2 CAR-T cells using CD28 or 4-1BB costimulatory domains and assessed their CAR expression, activation phenotype, subset composition, and cytotoxicity. We then engineered three bicistronic dual-target CARs with distinct CD3ζ configurations and evaluated their functionality through *in vitro* assays and *in vivo* NUGC4-Luc gastric cancer xenograft models.

**Results:**

The single-target CLDN18.2-CD28 CAR-T cells exhibited the strongest cytotoxicity *in vitro*. Among the bicistronic designs, the double-CD3ζ CAR-T cells showed superior expansion, differentiation, and cytotoxicity compared to alternative bicistronic constructs and single-target CAR-T cells. Bulk RNA sequencing revealed enhanced immune activation and reduced exhaustion markers in the double-CD3ζ CAR-T cells. In the NUGC4-Luc xenograft model, the double-CD3ζ CAR-T cells achieved a tumor growth inhibition of 63.23%, outperforming the 48.06% inhibition observed with single CLDN18.2 CAR-T cells.

**Conclusions:**

The study identifies a bicistronic dual-target, double-CD3ζ CAR-T design that enhances T-cell functional fitness and antitumor efficacy in gastric cancer models. This approach offers a promising strategy for addressing antigen heterogeneity and improving the durability of CAR-T therapies in solid tumors.

## 1 Introduction

Gastric cancer remains a major cause of cancer-related mortality worldwide, and therapeutic options for advanced disease are still limited (1). Although immunotherapies such as immune checkpoint inhibitors have improved outcomes, clinical responses remain inconsistent due to tumor heterogeneity, an immunosuppressive tumor microenvironment (TME), and antigen escape (2).

Chimeric antigen receptor (CAR) T-cell therapy has transformed the treatment of hematologic malignancies, yet its efficacy in solid tumors, including gastric cancer, is hindered by restricted tumor infiltration, suppressive myeloid populations, and unstable antigen expression (3). These limitations highlight the need for optimized CAR designs capable of improving persistence, enhancing antigen recognition, and resisting TME-induced dysfunction.

PD-L1 and Claudin18.2 (CLDN18.2) have emerged as compelling targets for gastric cancer. PD-L1 contributes to immune evasion and is frequently upregulated in gastric tumors (4, 5), while CLDN18.2 shows high tumor selectivity and stable expression in a substantial proportion of gastric cancers (6, 7). The distinct features of these targets highlight the importance of investigating CAR-T cells that target both PD-L1 and CLDN18.2. However, the optimal design of CAR-T cells targeting these antigens, particularly with regard to the choice between CD28 and 4-1BB costimulatory domains, remains unclear (8). A systematic comparison is needed to understand how structural features influence T-cell activation, persistence, and antitumor efficacy.

To address these challenges, dual-targeting CAR strategies have emerged as a potential solution. By broadening antigen coverage, dual-target CARs can help overcome tumor heterogeneity and mitigate antigen-loss escape (9). These constructs can be engineered with various intracellular signaling architectures, including designs containing either a single CD3ζ chain or two CD3ζ modules. These variations can influence signaling strength, persistence, and susceptibility to exhaustion (10). However, it is unclear whether dual-CAR systems, irrespective of CD3ζ configuration, outperform the most effective single-CAR constructs in gastric cancer.

In this study, we aim to systematically compare CAR designs targeting PD-L1 and CLDN18.2, incorporating CD28 or 4-1BB costimulatory domains in single CAR-T constructs and evaluating bicistronic dual CAR-T systems with distinct CD3ζ configurations. Our first objective is to identify the most effective single-CAR design based on cytotoxicity and T-cell functional performance. We then assess whether dual-CAR architectures, including bicistronic constructs containing one or two CD3ζ domains, offer enhanced antitumor activity. This comparative engineering approach will help define how antigen specificity, signaling-domain composition, and CAR structural organization influence T-cell responses and guide the development of next-generation CAR-T therapies for gastric cancer.

## 2 Methodology

### 2.1 Cell lines and Cell culture

The human embryonic kidney cell line HEK293T was obtained from the National Collection of Authenticated Cell Cultures (China) and maintained in Dulbecco’s Modified Eagle Medium (DMEM; Beyotime, China) supplemented with 10% heat-inactivated fetal bovine serum (FBS; Gibco, USA) at 37 °C in a humidified incubator with 5% CO_2_.

The human gastric cancer cell line NUGC4 and human peripheral blood mononuclear cells (PBMCs) were purchased from Shanghai Yayou Biotechnology Co., Ltd. NUGC4 cells were cultured in RPMI-1640 medium (Beyotime, China) containing 10% heat-inactivated FBS.

T cells were purified by negative selection using the EasySep™ Human T Cell Isolation Kit (STEMCELL Technologies, USA). Purified T cells were activated with Dynabeads™ Human T-Activator CD3/CD28 (Thermo Fisher Scientific, USA) and cultured in RPMI-1640 supplemented with 10% heat-inactivated FBS, recombinant human IL-2, IL-7, and IL-15 (R&D Systems, USA). After 48 h, activation beads were removed and T cells were transduced with lentiviral supernatants at a multiplicity of infection (MOI) of 20. Medium was replaced every 2 days, and CAR-T cells were expanded for 12–14 days before downstream assays.

### 2.2 Assessment of CAR-T cell expansion

CAR-T cell expansion was monitored over a 12-day culture period following activation and transduction. Fresh medium was replenished as needed, and viable cell numbers were quantified every 2 days using cell counting. Proliferation was expressed as fold expansion, calculated as:


$$Fold\;expansion=\frac{N_{t}}{N_{0}},$$

$N_{t}$ represents the number of viable cells at time point, while $N_{0}\;$ denotes the number of viable cells at the initial time point.

### 2.3 Lentiviral vector construction

The lentiviral vectors for PD-L1 and CLDN18.2 CAR constructs were based on the GV401 backbone (GeneChem, Shanghai). The PD-L1-specific scFvs were derived from the Chinese patent CN107326014B. The CLDN18.2-binding scFv sequence was provided by CARsgen Therapeutics (Shanghai, China) (11) and is consistent with previously reported sequences.

All CAR cassettes were cloned into the GV401 backbone by restriction enzyme digestion and ligation, followed by verification through Sanger sequencing. In all bicistronic constructs, the PD-L1 CAR cassette was positioned upstream of the CLDN18.2 CAR cassette for cloning convenience, and this orientation was kept identical across all bicistronic designs to ensure consistency in comparative evaluation of different CD3ζ configurations.

### 2.4 PD-L1 and CLDN18.2 double staining of CAR-T cells

For double staining of PD-L1 and CLDN18.2 on CAR-T cells, recombinant PD-L1 (Sino Biological, China) and CLDN18.2 proteins (Acro Biosystems, USA) were used as probes. After 48 hours of CAR-T cell expansion, the cells were harvested, washed with PBS, and resuspended at 1 × 10^6^ cells per tube. To minimize non-specific binding, cells were pre-incubated with Fc block (BD Biosciences, USA) for 15 minutes at 4 °C. Subsequently, PD-L1 and CLDN18.2 probes were added simultaneously to specifically bind to the CAR scFv regions. After incubation, cells were washed three times with PBS and resuspended in PBS supplemented with 2% FBS. Flow cytometric analysis was performed, and data were analyzed using FlowJo software.

### 2.5 Flow cytometry

Cells were collected, washed with PBS, and resuspended at 1 × 10^6^ cells per tube. Cell viability was assessed by staining with a fixable viability dye (BD, US) for 15 minutes at 4 °C. After viability staining, cells were incubated with a pre-mixed surface antibody cocktail for 30 minutes at 4 °C in the dark. The antibody panel consisted of fluorochrome-conjugated monoclonal antibodies against human CD3, CD4, CD8, CD45RA, CCR7, PD-1, LAG-3, TIM-3, CD25, CD38, HLA-DR, and the G4S linker (BD, US). Cells were then washed three times with PBS and resuspended in PBS supplemented with 2% FBS.

Surface expression of PD-L1 and CLDN18.2 on NUGC4 cells was assessed using fluorochrome-conjugated monoclonal antibodies against human PD-L1 (BD, USA) and Claudin-18.2 (Novus Biologicals, USA), with isotype controls (BD, USA) to validate specificity.

For intracellular staining, cells were permeabilized using Permeabilization Buffer (BD, USA), followed by incubation with antibodies targeting TOX (Thermo Fisher, USA) and TCF7 (BD, USA) for 30 minutes at 4 °C. After washing, cells were resuspended in PBS supplemented with 2% FBS for flow cytometry analysis.

Samples were acquired on a flow cytometer (Thermo Fisher Scientific, USA), and data were analyzed using FlowJo software (BD, US).

### 2.6 *In vitro* coculture cytotoxicity assay

The cytotoxic activity of different CAR-T constructs against NUGC4 gastric cancer cells was evaluated using a luciferase-based assay. NUGC4 cells were seeded in 96-well white flat-bottom plates. CAR-T cells were then added at different E:T ratios.

After 6, 12, 18, and 24 h of coculture, cytotoxicity was quantified using the KeyTec^®^ Ultra Luciferase Detection Kit (VKEYBIO, China) according to the manufacturer’s protocol. Relative light units (RLUs) were recorded for each well, and background signal from blank wells was subtracted. Specific lysis was calculated as:


Lysis(%)=(Dexp−Dblank)/(Dmax−Dblank)×100%

Where:

*Dmax* is the maximum lysis value; *Dblank* is the background value; *Dexp* is the experimental lysis value.

### 2.7 Bulk RNA sequencing

Total RNA extraction and stranded mRNA library construction were performed by Novogene (Beijing, China) using the TruSeq Stranded mRNA Library Preparation Kit (Illumina, US), following the manufacturer’s instructions. Sequencing was conducted on an Illumina HiSeq 4000 platform with paired-end reads. Downstream analyses were performed in R (version 3.6.2). Read counts were normalized, and differential gene expression analysis was carried out using the DESeq2 package.

### 2.8 *In vivo* experiments

All animal procedures were approved by the Institutional Animal Ethics Committee of Shanghai Tenth People’s Hospital (SHDSYY-2025-P0085) and followed the institution’s Animal Care and Use Guidelines. Regular health monitoring was conducted throughout the study. Mice were euthanized immediately when they met humane endpoint criteria, including signs of severe distress, ≥20% body-weight loss from baseline, rapid body-weight loss judged by veterinary staff, ulceration or impaired mobility, or tumor volume approaching 2,000 mm³. For survival analysis, euthanasia due to humane endpoint criteria was counted as an event. Tumor-volume and body-weight measurements after euthanasia were not included in longitudinal analyses.

Subcutaneous tumors were established by injecting 5 × 10^6^ NUGC4-Luc cells into the left axillary subcutaneous space. Tumor size was measured every 3 days including length (L) and width (W). Volume (V) was calculated using:


$$V=0.5\times L\times W^{2}.$$

Mice were intravenously injected with 3 × 10^6^ CAR-positive T cells. Bioluminescence imaging (BLI) was performed on Days 0, 7, 14, and 21. Images were acquired using an AniView100 system (Berthold Technologies, UK).

Tumor growth inhibition (TGI) at Day 21 was determined as:


$$TGI(\%)=(1\frac{R\bar{T}V_{\text{treat}}}{R\bar{T}V_{\text{control}}})\times 100\%$$

Relative tumor volume (RTV) was calculated as:


$$RTV=\frac{Vt}{V0}$$

where V_t_ represents the tumor volume at time point t, and V_0_ represents the tumor volume at Day 0.

Body weight change was calculated as:


$$\text{Body-weight change}(\%)=\frac{BW_{t}-BW_{0}}{BW_{0}}\times 100\%$$

$BW_{0}\;and\;BW_{t}\;$ represent body weight on Day 0 and at time t, respectively.

### 2.9 Statistical analysis

Data were analyzed using FlowJo for flow cytometry and R (v3.6.2) for downstream analyses. All *in vitro* experiments were performed with at least three independent biological replicates (n = 3) unless otherwise specified. For *in vivo* experiments, group sizes (n) are indicated in the corresponding figure legends. Results are presented as mean ± SD. Two-group comparisons used unpaired two-tailed Student’s t-tests. For ≥3 groups, one-way ANOVA with *post hoc* multiple-comparisons correction was applied; for experiments with two factors, two-way ANOVA with multiple-comparisons correction was used. Survival was analyzed by Kaplan–Meier method with log-rank (Mantel–Cox) test. Bulk RNA-seq differential expression was performed using DESeq2 with Benjamini–Hochberg FDR correction. P < 0.05 was considered statistically significant. Significance was denoted as ns (not significant), P < 0.05 (*), P < 0.01 (**), P < 0.001 (***), and P < 0.0001 (****).

## 3 Results

### 3.1 Functional characterization of PD-L1 and CLDN18.2-Targeted CAR-T cells with CD28 and 4-1BB costimulatory domains

**Figure 1:**
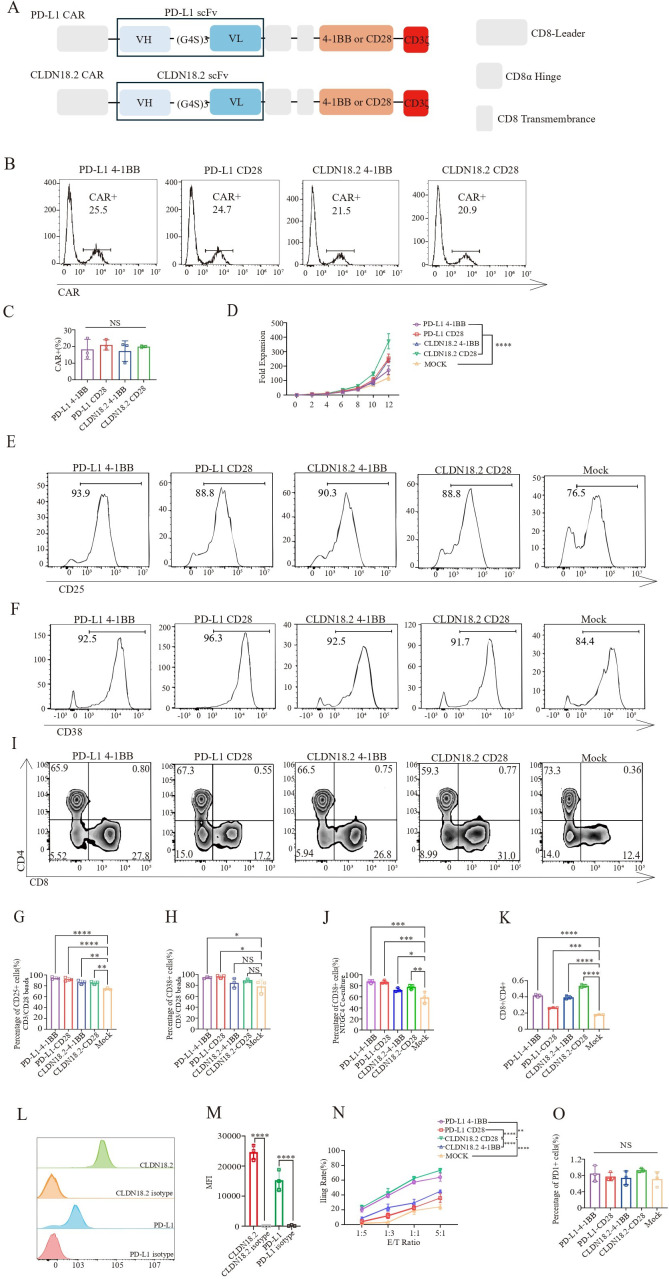
Functional evaluation of PD-L1- and CLDN18.2-targeted CAR-T cells with distinct costimulatory domains. **(A)** Schematic design of single-target CARs recognizing PD-L1 or CLDN18.2, incorporating CD28 or 4-1BB costimulatory domains. CAR surface expression in transduced T cells assessed by flow cytometry. Representative plots **(B)** and quantification of CAR-positive cells **(C)**. **(D)** Expansion kinetics of CAR-T cells during a 12-day culture period, shown as fold expansion relative to day 0. Early activation of CAR-T cells following CD3/CD28 stimulation, measured by CD25 **(E)** and CD38 **(F)** expression at 48 h, with quantification shown in **(G, H)**, using CAR-T cells from three independent donors. CD4^+^ and CD8^+^ T-cell subset distribution shown as representative plots **(I)** and summarized ratios **(K)**. **(J)** CAR-T cells were co-cultured with NUGC4 cells, and CD38 expression was measured. (**L, M**) Surface expression and MFI quantification of PD-L1 and CLDN18.2 on NUGC4 gastric cancer cells, validated by corresponding isotype controls. **(N)** *In vitro* cytotoxicity of CAR-T cells against NUGC4 targets measured by a luciferase-based killing assay at the indicated E:T ratios. Asterisks indicate comparisons of CLDN18.2-CD28 CAR-T cells versus PD-L1-4-1BB, PD-L1-CD28, CLDN18.2-4-1BB, and mock T cells at the indicated E:T ratios. **(O)** PD-1 expression on CAR-T cells on day 7 post-activation. Data are presented as mean ± SD from n = 3 independent experiments. Statistical analyses were performed using one-way or two-way ANOVA with multiple-comparisons correction, as appropriate. Significance was defined as NS, not significant; *P < 0.05; **P < 0.01; ***P < 0.001; ****P < 0.0001.

We first generated a panel of CAR constructs targeting PD-L1 or CLDN18.2 incorporating either CD28 or 4-1BB costimulatory domains. Schematic diagrams illustrate the modular organization of each design (**Figure 1A**). Flow-cytometric analysis confirmed CAR surface expression across all constructs (**Figures 1B, C**), and CAR-positive T cells were subsequently enriched by flow cytometric sorting based on G4S linker detection. The proliferation analysis confirmed a faster expansion rate than the control group for all CAR-T constructs (**Figure 1D**).

To assess early T-cell activation, CAR-T cells were first stimulated with CD3/CD28 beads as a measure of their general activation responsiveness. Flow cytometric analysis revealed a marked increase in CD25 expression in both PD-L1- and CLDN18.2-targeted CAR-T cells compared with the empty vector-transduced mock T-cell group, indicating robust activation and upregulation of the IL-2 receptor α-chain (**Figures 1E, G**). Notably, CD38 upregulation was most pronounced in PD-L1-4-1BB and PD-L1-CD28 CAR-T cells after 48 h (**Figures 1F, H**). To assess antigen-dependent activation, CAR-T cells were co-cultured with NUGC4 cells. CD38 expression increased in all CAR-T groups (**Figure 1J**), consistent with the trend observed after CD3/CD28 stimulation.

Representative CD4/CD8 contour plots demonstrated that all CAR-T products contained both CD4^+^ and CD8^+^ subsets, with composition varying by CAR design (**Figures 1I, K**). PD-L1-4-1BB and CLDN18.2-4-1BB CAR-T cells displayed a relatively balanced distribution with modest CD8^+^ enrichment, PD-L1-CD28 CAR-T cells contained a higher proportion of CD4^+^ cells, and CLDN18.2-CD28 CAR-T cells showed a marked accumulation of CD8^+^ cells in the CD8^+^CD4^+^ quadrant. This pattern reflects stronger CD8-biased activation by CLDN18.2 in the context of CD28 costimulation, which preferentially drives CD8^+^ expansion and differentiation. In contrast, PD-L1–targeted CD28 CARs may experience weaker and/or fratricidal signaling due to PD-L1 expression on activated T cells, leading to a relative loss of CD8^+^ cells.

The cytotoxic capacity of the engineered CAR-T cells was then evaluated in standard effector-to-target (E/T) killing assays using NUGC4 gastric cancer cells. Isotype control staining confirmed the specific surface expression of PD-L1 and CLDN18.2 on NUGC4 cells, with rightward shifts and increased MFI ratios relative to controls (**Figures 1L, M**). CLDN18.2-CD28 CAR-T cells exhibited significantly higher cytotoxicity than other constructs across E/T ratios (**Figure 1N**). This superior killing likely reflects the CD8-skewed composition of the CLDN18.2-CD28 CAR-T together with CD28-driven strong early activation upon antigen engagement.

To investigate potential exhaustion, PD-1 expression was quantified on day 7 post-activation to assess potential T-cell exhaustion. The frequency of PD-1^+^ cells did not differ significantly between CAR-T groups, suggesting that neither the target antigen nor the costimulatory domain influenced PD-1 expression under these conditions (**Figure 1O**).

### 3.2 Construction and characterization of Bicistronic CAR-T cells targeting CLDN18.2 and PD-L1

**Figure 2:**
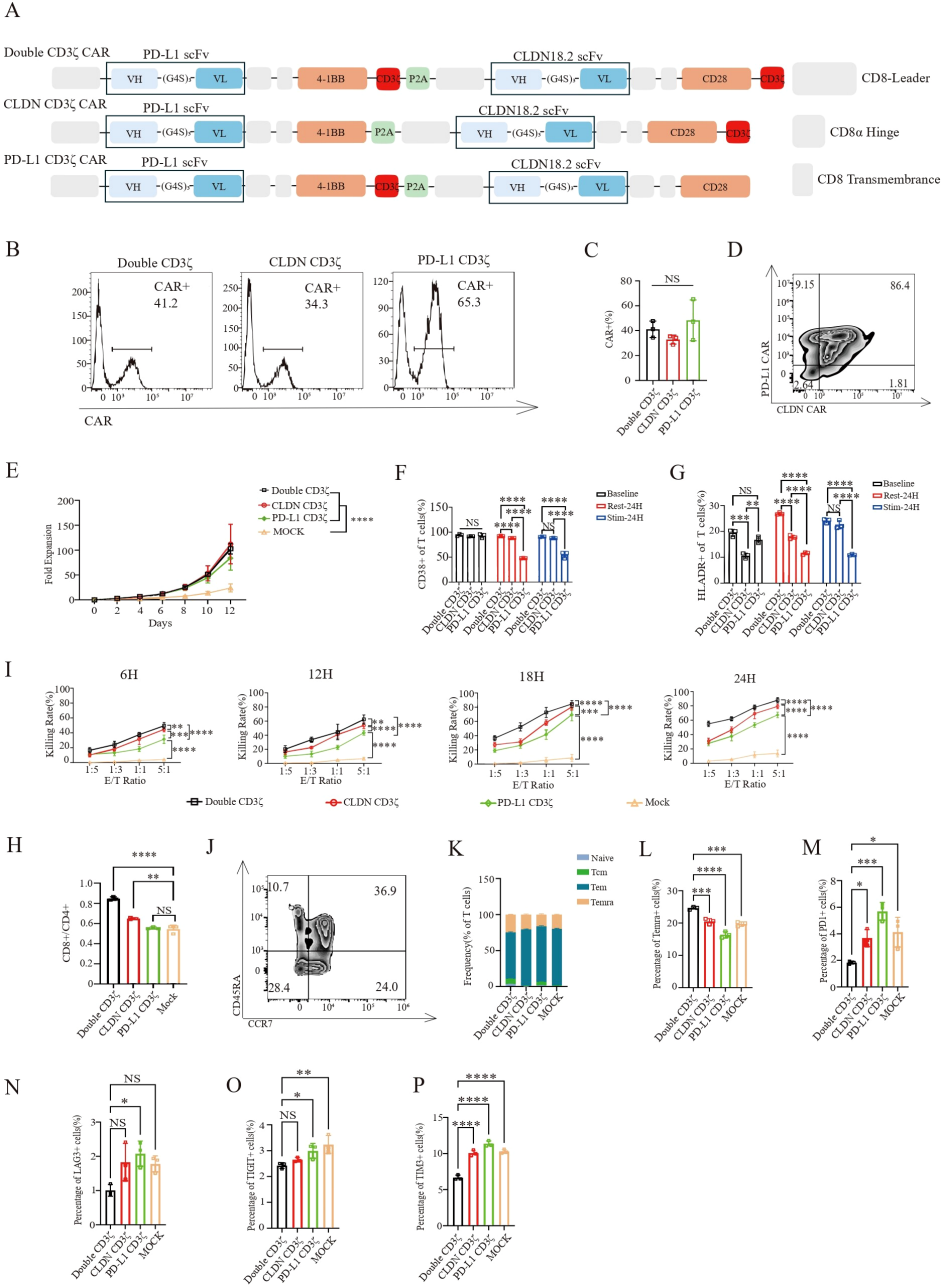
Construction and phenotypic characterization of bicistronic CLDN18.2/PD-L1 CAR-T cells with distinct CD3ζ placement. **(A)** Schematics of three bicistronic constructs (double CD3ζ, CLDN-CD3ζ, and PD-L1-CD3ζ). **(B, C)** CAR surface expression at day 2 post-transduction. **(D)** Flow cytometric validation of dual CAR surface expression in bicistronic CAR-T cells. **(E)** Expansion kinetics. **(F, G)** HLA-DR and CD38 expression at baseline (48 h post-transduction), after 24 h rest, or after 24 h co-culture with NUGC4 cells. **(H)** CD8/CD4 ratio. **(I)** Cytotoxicity of bicistronic CAR-T cells against target cells at the indicated E:T ratios and co-culture durations. **(J)** Representative flow cytometric gating strategy for T-cell differentiation subsets based on CCR7 and CD45RA expression, defining naïve T cells, Tcm, Tem, and Temra. **(K, L)** Quantification of T-cell differentiation subsets, with emphasis on the proportion of Temra cells among the three bicistronic CAR-T cell products. **(M–P)** Expression of immune checkpoint receptors LAG-3, PD-1, TIGIT, and TIM-3 on bicistronic CAR-T cells at day 7 after activation. Data are presented as mean ± SD from 3 independent experiments. Statistical significance was determined by one-way ANOVA or two-way ANOVA with appropriate multiple-comparisons testing. NS, not significant; *P < 0.05; **P < 0.01; ***P < 0.001; ****P < 0.0001.

Building on cytotoxicity assays of monospecific CARs, in which CLDN18.2-CD28 and PD-L1-4-1BB variants exhibited superior lysis of NUGC4 cells (**Figure 1L**), we developed three bicistronic CAR constructs targeting both CLDN18.2 and PD-L1 to enhance efficacy against gastric tumors. **Figure 2A** shows three bicistronic CARs with distinct intracellular signaling configurations: double CD3ζ, single CLDN CD3ζ, and single PD-L1 CD3ζ. In all constructs, the PD-L1 CAR is positioned upstream of the CLDN18.2 CAR for cloning convenience, separated by a P2A sequence, and both CAR modules include CD8 leader, hinge, and transmembrane domains. The various CD3ζ placements were strategically engineered to optimize ITAM phosphorylation for synergistic activation and to evaluate whether they could reduce T-cell exhaustion and enhance cytotoxicity within heterogeneous tumors.

Flow cytometry analysis at day 2 post-transduction revealed CAR surface expression across engineered groups (**Figures 2B, C**). To further verify simultaneous surface expression of both CAR molecules in the bicistronic constructs, CAR-T cells were first enriched by cell sorting and then analyzed by flow cytometry using recombinant human PD-L1 and Claudin-18.2 probes, which identified a double-positive population in bicistronic CAR-T cells (**Figure 2D**).

*In vitro* expansion assays further demonstrated increased proliferation of all three bicistronic CAR-T cell products compared with empty vector-transduced T-cell mock group, indicating that the optimized signaling configurations support *in vitro* expansion during the 12-day culture period (**Figure 2E**).

To assess the activation status of engineered T cells, CD38 and HLA-DR expression were quantified by flow cytometry in three CD3ζ-based CAR-T products at baseline (48 h post–lentiviral transduction), after a 24 h resting period in the absence of target cells, and following 24 h co-culture with NUGC4 cells. HLA-DR and CD38 expression did not increase uniformly after 24 h of culture across all groups, indicating construct-dependent activation patterns. Notably, double CD3ζ CAR-T cells exhibited significantly higher HLA-DR and CD38 expression after 24 h of unstimulated culture compared with the other constructs. In contrast, CLDN–CD3ζCAR-T cells showed moderate increases, whereas PD-L1 CD3ζ CAR-T cells displayed comparatively lower expression levels. Following 24 h co-culture with NUGC4 cells, these differences were attenuated, and no consistent significant differences were observed between the double CD3ζ and CLDN–CD3ζ groups (**Figures 2F, G**).

To determine whether intracellular signaling configuration influences CAR-T subset composition and effector function, we compared three bicistronic CAR-T designs. The double CD3ζ construct displayed the highest CD8^+^/CD4^+^ ratio (**Figure 2H**). In cytotoxicity assays, target-cell killing increased with E/T ratios and prolonged co-culture from 6 to 18 h, with no further improvement at 24 h. Notably, double CD3ζ CAR-T cells consistently mediated the greatest cytotoxicity across all time points, in line with their elevated CD8^+^/CD4^+^ ratio (**Figure 2I**).

We next assessed the differentiation status of the three bicistronic CAR-T products. T-cell subsets were defined by CCR7 and CD45RA expression, classifying cells as naïve T cells, central memory T cells (Tcm), effector memory T cells (Tem), and terminally differentiated effector memory T cells (Temra) (**Figure 2J**). Across all groups, the double CD3ζ construct yielded the highest proportion of Temra cells (**Figures 2K, L**), a subset characterized by elevated expression of cytotoxic effector molecules such as granzyme and perforin and associated with enhanced cytolytic capacity.

We next assessed immune checkpoint receptor expression on the three bicistronic CAR-T constructs at day 7 post-activation. As shown in **Figures 2M–P**, double CD3ζ CAR-T cells exhibited lower levels of LAG-3, PD-1, TIGIT, and TIM-3 compared with CLDN-CD3ζ and PD-L1-CD3ζ CAR-T cells, with significant reductions observed for PD-1 (**Figure 2O**) and TIM-3 (**Figure 2P**). These results indicate that dual CD3ζ signaling promotes a more robust and durable T-cell activation, helping maintain effector function while limiting exhaustion.

### 3.3 Enhanced differentiation, cytotoxicity, and immune activation of double CD3ζ CAR-T cells compared to CLDN18.2 CAR-T cells

**Figure 3:**
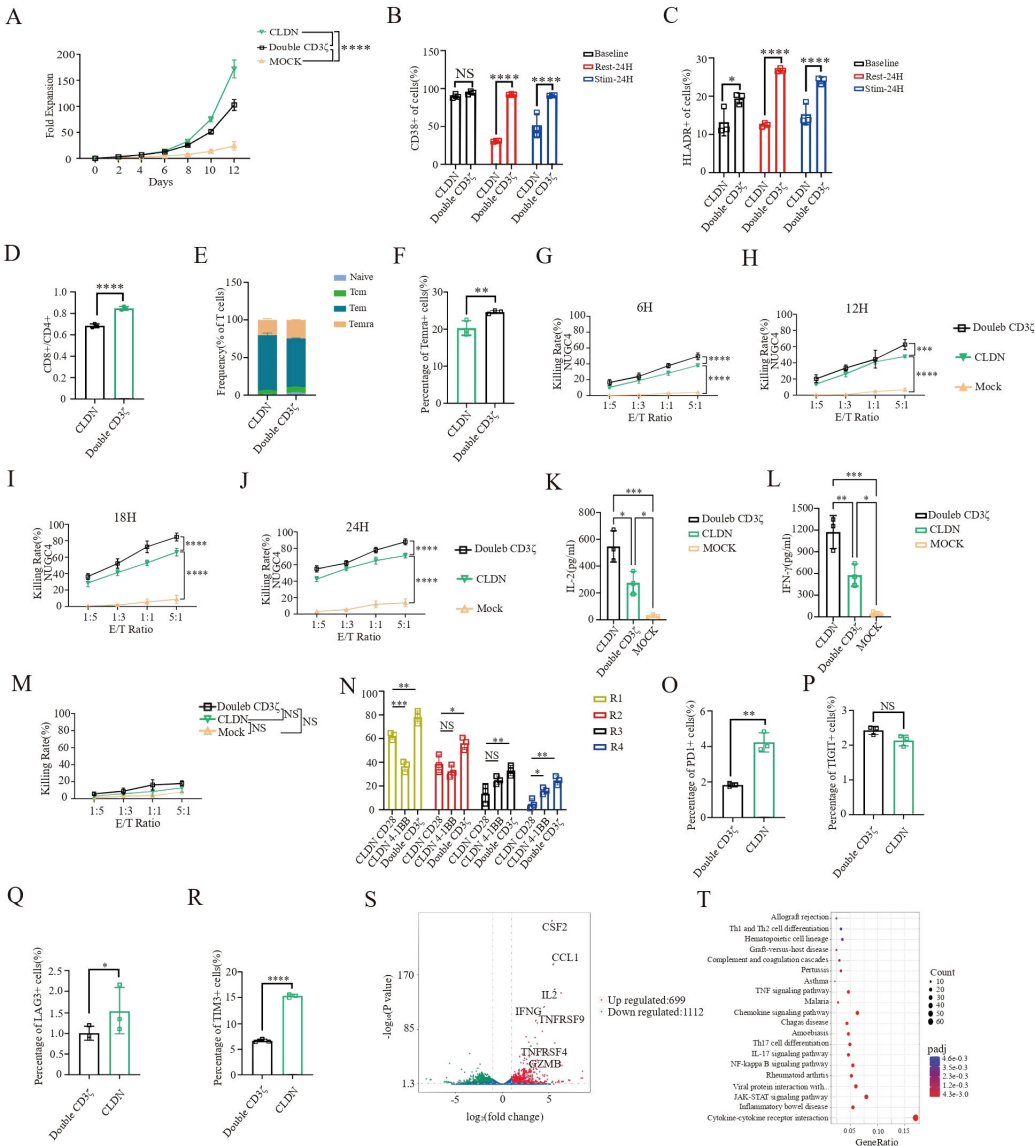
Double CD3ζ CAR-T cells show enhanced activation persistence, cytotoxicity, and immune transcriptional programs compared with CLDN18.2 CAR-T cells. **(A)** Fold expansion during 12 days of culture. **(B, C)** Flow-cytometric analysis of CD38 and HLA-DR expression at baseline, after 24 h rest, or after antigen re-challenge. **(D)** CD8^+^/CD4^+^ ratio. **(E, F)** T-cell differentiation subsets and Temra frequency. **(G–J)** Luciferase-based cytotoxicity against NUGC4 target cells at indicated E:T ratios after 6, 12, 18, and 24 h. **(K, L)** IL-2 and IFN-γ secretion after 24 h co-culture with tumor cells. **(M)** Cytotoxicity of CAR-T cells against NUGC4 targets in the presence of PD-L1 and CLDN18.2 blocking antibodies. **(N)** Cytotoxicity during repeated antigen stimulation. **(O–R)** Expression of exhaustion-associated markers PD-1, TIGIT, LAG-3, and TIM-3 after repeated stimulation. **(S)** Differentially expressed immune-related genes in double CD3ζ versus CLDN18.2 CAR-T cells. **(T)** Pathway enrichment analysis of upregulated genes in double CD3ζ CAR-T cells. Data are presented as mean ± SD from n = 3 biological replicates. Statistical analyses were performed as described in the Methods, using unpaired two-tailed Student’s t test for two-group comparisons and one-way or two-way ANOVA with multiple-comparisons correction where appropriate. Significance is indicated as NS, not significant; *P < 0.05; **P < 0.01; ***P < 0.001; and ****P < 0.0001.

To determine whether the double-CAR architecture outperformed the single-CAR design, we compared expansion, activation, differentiation, CD8^+^/CD4^+^ ratio, and cytotoxicity between CLDN18.2 CAR-T and double CD3ζ CAR-T cells over a 12-day culture period. CLDN18.2 CAR-T cells showed greater fold expansion than double CD3ζ CAR-T cells (**Figure 3A**). Despite this difference, double CD3ζ CAR-T cells exhibited higher CD38 expression after a 24 h resting period and upon antigen re-challenge, consistent with enhanced signaling persistence and a greater capacity for rapid reactivation (**Figure 3B**). HLA-DR expression increased in both groups after antigen re-challenge, confirming preserved secondary responsiveness. However, at the resting time point, HLA-DR expression remained modestly lower in double CD3ζ CAR-T cells than in CLDN18.2 CAR-T cells, suggesting distinct activation kinetics, with earlier peak activation in the double CD3ζ group and a more delayed, sustained response in the CLDN18.2 group (**Figure 3C**). Because activation-marker changes after target-cell co-culture were relatively small, we further assessed PD-L1 expression and cell viability after 24 h of stimulation (**Supplementary Figures 1A, B**). Neither parameter differed significantly among groups, arguing against PD-L1 upregulation, fratricide, or overt toxicity. Instead, the limited increase after NUGC4 co-culture is more consistent with a ceiling effect resulting from the pre-activated state of the CAR-T products together with tonic signaling associated with the dual CD3ζ architecture.

The double CD3ζ CAR-T cells exhibited a significantly higher CD8^+^/CD4^+^ ratio compared to the CLDN CAR-T cells (**Figure 3D**). Regarding differentiation, the double CD3ζ CAR-T cells had a significantly higher percentage of Temra cells compared to the CLDN CAR-T cells (**Figures 3E, F**). In short-term cytotoxicity assays against NUGC4 cells, double CD3ζ CAR-T cells consistently demonstrated higher killing efficiency than CLDN CAR-T cells at all E/T ratios and across all time points (6h, 12h, 18h, and 24h) (**Figures 3G–J**). To further examine whether the Temra-enriched phenotype was associated with stronger effector function, we measured cytokine secretion in the supernatants after 24 h of co-culture with tumor cells. Double CD3ζ CAR-T cells secreted significantly higher levels of IL-2 and IFN-γ than CLDN18.2 CAR-T cells, whereas empty vector-transduced T-cell mock group showed minimal cytokine production (**Figures 3K, L**). These findings suggest that, although double CD3ζ CAR-T cells did not exhibit greater expansion than CLDN18.2 CAR-T cells, they displayed a more favorable CD8^+^ T-cell phenotype and superior cytotoxic function compared with the single CAR construct.

To further determine whether the enhanced cytotoxicity of double CD3ζ CAR-T cells was dependent on recognition of both PD-L1 and CLDN18.2, we evaluated CAR-T activity in both isogenic and heterogeneous antigen settings. In NUGC4 cells, antibody-blocking cytotoxicity assays demonstrated that simultaneous blockade of PD-L1 and CLDN18.2 resulted in a greater attenuation of tumor-cell killing, reducing cytotoxicity to levels comparable to background, thereby supporting the contribution of both target antigens to CAR-T activity (**Figure 3M**). In parallel, a 24 h killing assay was performed using CLDN18.2/PD-L1-positive NUGC4 cells, CLDN18.2/PD-L1-double-negative HGC27 cells, mixed cultures (50% HGC27 + 50% NUGC4), and the intermediate-antigen-expression gastric cancer cell line NCI-N87. Double CD3ζ CAR-T cells showed enhanced killing in NUGC4, mixed, and NCI-N87 cultures, but not in HGC27-only cultures, further supporting improved activity in heterogeneous tumor settings (**Supplementary Figures 1C, D**).

During repeated antigen stimulation every 24 h, cytotoxicity declined in all groups, but double CD3ζ CAR-T cells retained the highest overall killing activity (**Figure 3N**). CLDN18.2-4-1BB CAR-T cells outperformed CLDN18.2-CD28 CAR-T cells at round 4, in line with the reported benefit of 4-1BB signaling under prolonged antigen exposure, although no significant difference was observed at rounds 2 and 3.

Consistent with this, after repeated stimulation, double CD3ζ CAR-T cells exhibited lower expression of exhaustion-associated markers, including PD-1, LAG-3, and TIM-3, whereas TIGIT expression was not significantly different between groups (**Figures 3O–R**). However, intracellular staining revealed no significant differences in TOX or TCF7 expression between double CD3ζ and CLDN18.2 CAR-T cells after 4 rounds of repeated antigen stimulation, suggesting that exhaustion- and stemness/persistence-associated transcriptional states were comparable under these conditions (**Supplementary Figures 1E, F**).

Gene expression analysis further revealed significant upregulation of immune-related genes, including TNFRSF4, CSF2, CCL1, IL-2, GZMB, TNFRSF9, and IFNG in double CD3ζ CAR-T cells (**Figure 3S**). Pathway analysis showed upregulation of key immune pathways such as the TNF signaling pathway, Th1/Th2 differentiation, and cytokine-cytokine receptor interactions (**Figure 3T**), indicating a more robust activation profile that supports enhanced T-cell activation and cytotoxicity.

### 3.4 *In vivo* antitumor activity of CAR-T cells in a gastric cancer xenograft model

**Figure 4:**
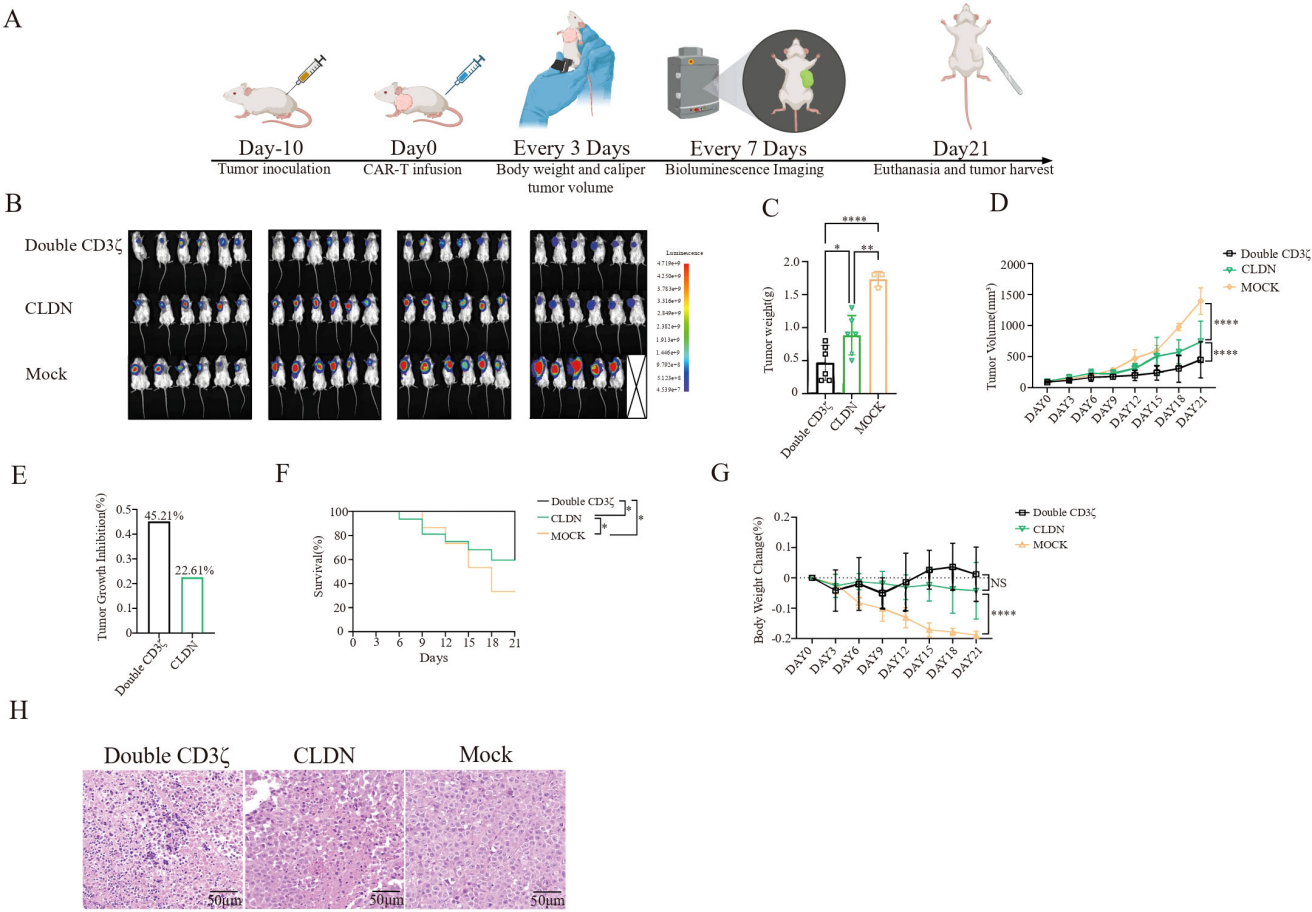
Double CD3ζ CAR-T cells exhibit enhanced antitumor activity in a gastric cancer xenograft model. **(A)** Experimental schema of the NUGC4-Luc subcutaneous xenograft model. **(B)** Representative longitudinal BLI images of mice treated with double CD3ζ CAR-T, CLDN CAR-T, or mock T cells. **(C)** Tumor weight at endpoint. **(D)** Tumor growth curves calculated from caliper-measured tumor volumes; measurements after euthanasia at humane endpoint **(E)** Tumor growth inhibition at day 21 relative to the mock group. **(F)** Kaplan–Meier survival analysis; euthanasia at humane endpoint counted as an event. **(G)** Percentage change in body weight over time; animals euthanized at ≥20% loss were not measured afterward. **(H)** H&E staining of tumors showing increased necrosis in the double CD3ζ CAR-T group. For **(F)**, n = 13 mice per group; the other analyses shown in this figure were performed using a separate cohort with n = 6 mice per group. Statistical analyses were performed using one-way or two-way ANOVA with multiple-comparisons correction where appropriate, unpaired two-tailed Student’s *t*-test, or log-rank (Mantel–Cox) test, as appropriate. NS, not significant; *P < 0.05; **P < 0.01; and ****P < 0.0001.

To evaluate the *in vivo* antitumor efficacy of CAR-T cells, subcutaneous gastric cancer xenografts were established on day −10, followed by CAR-T cell infusion on day 0. Tumor growth was monitored by body weight and tumor volume measurements every 3 days. Tumor burden was assessed using weekly live imaging, and tumors were harvested on day 21 for downstream analyses (**Figure 4A**).

Weekly live imaging from weeks 0 to 3 revealed that the Double CD3ζ CAR-T group exhibited the lowest bioluminescent tumor signal, indicating the most effective tumor control. In contrast, the empty vector-transduced T-cell mock group showed progressive tumor growth, while the CLDN CAR-T group displayed intermediate suppression (**Figure 4B**).

At the study endpoint (day 21), tumor weight measurements showed a significant reduction in the Double CD3ζ CAR-T group compared to both the CLDN CAR-T and Mock groups (**Figure 4C**). Tumor volume measurements further confirmed the efficacy of Double CD3ζ CAR-T therapy, with a significant inhibition of tumor growth, compared to both CLDN and Mock treatments (**Figure 4D**). The Double CD3ζ CAR-T group demonstrated a 63.23% inhibition of tumor growth, whereas the CLDN CAR-T group showed 48.06% inhibition (**Figure 4E**). These findings were further supported by the survival analysis, which showed improved survival in mice treated with Double CD3ζ CAR-T cells (**Figure 4F**).

Throughout the study, mice treated with Double CD3ζ CAR-T and CLDN CAR-T maintained stable body weight. In contrast, the Mock group exhibited significant weight loss after day 12 (**Figure 4G**). HE staining of excised tumors further showed more extensive necrotic regions in the Double CD3ζ CAR-T group compared with the CLDN CAR-T and mock groups (**Figure 4H**).

## 4 Discussion

CLDN18.2 has emerged as a clinically actionable antigen in advanced gastric and gastroesophageal junction adenocarcinoma due to its tumor-restricted surface expression and substantial prevalence in biomarker-selected patients (12, 13). Its clinical relevance is supported by the phase III SPOTLIGHT and GLOW trials, in which the addition of zolbetuximab to first-line chemotherapy significantly improved survival in CLDN18.2-positive, HER2-negative disease (14, 15). These findings suggested the importance of CLDN18.2 CAR-T design for gastric cancer immunotherapy.

However, the rational engineering of CAR-T cells for solid tumors requires balancing potent antitumor activity with durability, resistance to exhaustion, and adaptability to tumor heterogeneity. In gastric cancer, this challenge is amplified by spatially heterogeneous antigen expression and an immunosuppressive tumor microenvironment (2). Accordingly, we adopted a stepwise engineering strategy: we first optimized single-target CAR constructs to identify an effective baseline design, and then advanced to a dual-target architecture with reinforced intracellular signaling to better address heterogeneity and functional attrition.

Consistent with this approach, our single-target screening identified CLDN18.2-CD28 as the most potent construct in cytotoxicity assays. Mechanistically, this likely reflects a combination of robust antigen engagement on CLDN18.2-positive tumor cells and CD28-driven rapid effector differentiation, which is typically associated with strong early cytotoxic activity and cytokine production (16–18). While such cytotoxic potency is advantageous, durable efficacy in solid tumors additionally requires resistance to immunosuppressive signals and sustained functionality within a hostile tumor microenvironment (19, 20). This trade-off emphasizes that selecting the most active single CAR is necessary but not sufficient, and supports the need for further structural optimization.

In this context, the choice of costimulatory domain is a key determinant of CAR-T cell behavior. CD28-based CARs are known to promote rapid expansion and strong early effector function, whereas 4-1BB signaling supports metabolic fitness, memory differentiation, and long-term persistence (21–23). In the present study, CD28- and 4-1BB-containing CAR-T cells showed no significant differences in early exhaustion marker expression, suggesting comparable short-term functional integrity. The superior cytotoxicity observed with CLDN18.2-CD28 CAR-T is therefore consistent with CD28-mediated effector programming (24). Nevertheless, whether this enhanced early activation translates into increased long-term exhaustion warrants further investigation.

Under repeated antigen stimulation, CLDN18.2-4-1BB CAR-T cells outperformed CLDN18.2-CD28 CAR-T cells at later rounds, especially round 4, consistent with the role of 4-1BB signaling in sustaining function during chronic antigen exposure (24). These findings suggest that costimulatory domains differentially influence early versus sustained antitumor activity.

Although CLDN18.2 provides a relatively tumor-selective anchor antigen, gastric cancer is marked by substantial intratumoral heterogeneity, including variable tumor-associated antigen expression and dynamic regulation of immune checkpoint molecules (25). This complexity provides a strong rationale for dual-target CAR-T approaches, which may broaden tumor recognition across heterogeneous cell populations and reduce the likelihood of antigen-loss escape (26, 27). Notably, optimized dual-target CAR-T designs have shown improved antitumor activity and reduced escape in solid tumor models (28). Within this context, PD-L1 is an attractive complementary target because it reflects an immune-evasive tumor state and may extend antigen coverage beyond CLDN18.2-positive compartments (29).

In this study, we primarily used NUGC4 gastric cancer cells, which are nearly 100% positive for both PD-L1 and CLDN18.2, to evaluate CAR-T cytotoxicity in vitro and in vivo. While this model provides a controlled system to assess dual-target CAR efficacy, it does not fully recapitulate the heterogeneous antigen distribution observed in human gastric tumors. In patient samples, PD-L1 and CLDN18.2 may not always be co-expressed on the same tumor cells; instead, PD-L1 can also be expressed on immune or stromal cells within the TME, while CLDN18.2 is largely tumor-specific. Such spatial and cellular heterogeneity may influence the functional activity and targeting efficiency of dual CAR-T cells. Future studies should use patient-derived xenografts or organoids with heterogeneous expression to better evaluate dual CAR-T efficacy and targeting across diverse tumor and stromal populations.

A key translational challenge for dual PD-L1/CLDN18.2 CAR-T therapy is on-target/off-tumor toxicity. PD-L1 is not tumor-specific and can be induced on activated T cells, creating a potential fratricide/self-limiting activation risk; preclinical studies also suggest PD-L1-targeted CAR effector cells can damage both malignant and non-malignant PD-L1-expressing cells (30). This concern is clinically relevant, as a phase I PD-L1 CAR-T study in non-small cell lung cancer (NSCLC) reported severe delayed pulmonary toxicity, supporting the possibility of off-tumor injury in normal tissues such as the lung and potentially the liver (31). In contrast, CLDN18.2 has a narrower therapeutic window but is physiologically expressed in normal gastric mucosa, making gastric mucosal injury/gastritis an expected risk. In the final phase I satri-cel/CT041 trial, gastric mucosal injuries occurred in 8.2% of patients, although the overall safety profile was manageable (32), and the subsequent randomized phase II study similarly supported manageable safety (33). Therefore, future development of our dual-target construct should incorporate toxicity-mitigation strategies such as affinity tuning, logic-gated CAR designs, suicide switches, and local or regional delivery to improve safety while preserving antitumor efficacy (26, 34).

The rationale for dual targeting was further supported across distinct antigen contexts. The optimized double CD3ζ CAR enhanced killing in NUGC4 cells co-expressing CLDN18.2 and PD-L1, as well as in mixed tumor cultures and NCI-N87 cells with intermediate antigen expression, but not in antigen-double-negative HGC27 cells. These findings suggest that its advantage depends on antigen availability, supporting broader target coverage rather than nonspecific cytotoxicity. Although the antibody-blocking assay in NUGC4 cells further supported antigen-dependent cytotoxicity of double CD3ζ CAR-T cells, this approach does not fully substitute for genetic validation in an isogenic model. CRISPR/Cas9-mediated knockout of PD-L1 and/or CLDN18.2 in NUGC4 cells would provide a more definitive assessment of the contribution of each antigen to CAR-T activity. This remains a limitation of the present study and an important direction for future investigation.

Beyond antigen selection, we systematically optimized intracellular signaling architecture to improve functional fitness. We compared bicistronic CAR designs differing in CD3ζ placement, including a dual-target construct containing two CD3ζ signaling modules. This design was informed by growing evidence that tyrosine-based activation motif (ITAM) multiplicity and signal-strength calibration critically influence CAR-T cell fate decisions, exhaustion susceptibility, and functional persistence (35). In particular, subthreshold or intermittent CAR signaling has been linked to dysfunctional activation trajectories and exhaustion, whereas appropriately reinforced signaling can sustain effector function without necessarily accelerating terminal dysfunction (36), consistent with these findings (37).

Among the dual-target CAR-T configurations evaluated in this study, the double CD3ζ design demonstrated the most favorable functional and phenotypic profile, characterized by enhanced cytotoxic activity, an increased CD8/CD4 ratio, enrichment of terminal effector populations, and reduced expression of inhibitory receptors compared with alternative CD3ζ architectures. A plausible mechanistic explanation is that increased immunoreceptor ITAM signaling capacity augments the probability of productive T-cell activation upon antigen engagement, thereby reducing subthreshold or intermittent signaling events that are known to promote T-cell dysfunction and exhaustion (38, 39). Consistent with this interpretation, previous studies have shown that modulation of ITAM multiplicity can fine-tune signaling strength and functional outputs of engineered receptors, highlighting CAR signal architecture as a critical determinant of antitumor potency and selectivity (40–42).

At first consideration, the finding that double CD3ζ CAR-T cells exhibited both increased Temra differentiation and reduced exhaustion marker expression appears paradoxical. However, terminal effector differentiation does not necessarily indicate exhaustion, as Temra cells can remain highly cytotoxic and functionally competent (43). In our study, the Temra-enriched double CD3ζ CAR-T cells also showed stronger tumor killing, higher IL-2 and IFN-γ secretion, and lower checkpoint expression after repeated stimulation, consistent with a highly active rather than exhausted state. This phenotype may reflect more efficient signaling by the double CD3ζ architecture, which promotes rapid effector differentiation while concurrently limiting exhaustion-associated pathways, for example through reduced NFAT-dependent dysfunction or altered metabolic programming, thereby restraining the activation states that drive chronic dysfunction (17, 44).

Although initial transduction efficiency was modest (**Figure 1B**), all functional assays were performed using sorted CAR-positive T cells, ensuring that results reflect intrinsic CAR-dependent activity rather than mixed populations.

Bulk RNA sequencing showed that double CD3ζ CAR-T cells upregulated key immune activation genes (including cytokine and cytotoxic effector pathways) and enriched immune signaling programs consistent with enhanced functional fitness. This molecular profile aligns with their improved killing capacity and reduced exhaustion marker expression. These transcriptional findings were consistent with repeated-stimulation assays, where double CD3ζ CAR-T cells maintained the strongest cytotoxicity and showed lower PD-1, LAG-3, and TIM-3 expression after repeated antigen exposure, suggesting reduced chronic stimulation–induced dysfunction.

*In vivo* experiment, across the 3-week observation window, the double CD3ζ dual-target CAR-T cohort consistently displayed the lowest bioluminescent signal, indicating the most effective suppression of viable tumor burden compared with both mock and the CLDN18.2 single-CAR comparator. At the day 21, there is a significant reduction in excised tumor weight and a more pronounced inhibition of tumor-volume expansion. Quantitatively, tumor growth inhibition favored the optimized dual-target design (TGI 63.23%) over the CLDN18.2 single CAR (TGI 48.06%). Importantly, body weight remained stable in both CAR-T–treated groups while progressive weight loss emerged in the mock group after day 12. Taken together, these *in vivo* findings reinforce the functional advantage of the optimized dual-target/double-CD3ζ architecture in controlling established gastric cancer xenografts, reflecting the more favorable *in vitro* functional profile that would be expected to improve tumor debulking under repeated antigen encounter.

Despite the encouraging activity of the optimized dual-target CAR, several limitations warrant consideration. First, functional validation was largely based on short-term *in vitro* assays using a limited set of targets, which may not capture the antigen heterogeneity and immune pressures present in patients. Second, efficacy was assessed in a subcutaneous NUGC4-Luc xenograft with relatively short follow-up, a setting that does not fully model orthotopic growth, metastasis, or the human tumor microenvironment. Third, potential donor-to-donor variability and manufacturing robustness were not systematically evaluated. In addition, PD-L1 targeting raises inherent safety concerns because PD-L1 can be expressed on activated T cells and normal tissues, creating potential risks of fratricide and off-tumor toxicity; dedicated safety assessments were not performed. Finally, bulk RNA-seq may obscure cellular heterogeneity, and future studies should incorporate higher-resolution approaches (e.g., single-cell profiling) to better define underlying mechanisms.

## 5 Conclusions

Taken together, our results indicate that antigen selection and intracellular signaling architecture jointly determine CAR-T performance in gastric cancer models. While CLDN18.2−CD28 provided the strongest single-target cytotoxicity, an optimized dual-target CLDN18.2/PD−L1 CAR with double CD3ζ signaling achieved superior functional profiles, reduced exhaustion marker expression, and improved antitumor activity *in vivo*. These findings support rational multi-antigen CAR design as a strategy to address antigen heterogeneity in gastric cancer and provide a framework for next-generation CAR-T optimization toward clinically durable efficacy.

## Data Availability

The bulk RNA-sequencing data presented in this study have been deposited in the NCBI Sequence Read Archive (SRA) repository under BioProject accession number PRJNA1469375. Other data supporting the findings of this study are included within the article and its **Supplementary Material**. Further inquiries can be directed to the corresponding author(s).

## References

[1] Sexton RE Al Hallak MN Diab M Azmi AS . Gastric cancer: a comprehensive review of current and future treatment strategies. Cancer Metastasis Rev. (2020) 39:1179–203. doi: 10.1007/s10555-020-09925-3. PMID: 32894370 PMC7680370

[2] Yasuda T Wang YA . Gastric cancer immunosuppressive microenvironment heterogeneity: implications for therapy development. Trends Cancer. (2024) 10:627–42. doi: 10.1016/j.trecan.2024.03.008. PMID: 38600020 PMC11292672

[3] Keshavarz A Salehi A Khosravi S Shariati Y Nasrabadi N Kahrizi MS . Recent findings on chimeric antigen receptor (CAR)-engineered immune cell therapy in solid tumors and hematological Malignancies. Stem Cell Res Ther. (2022) 13:482. doi: 10.1186/s13287-022-03163-w. PMID: 36153626 PMC9509604

[4] Lin C He H Liu H Li R Chen Y Qi Y . Tumour-associated macrophages-derived CXCL8 determines immune evasion through autonomous PD-L1 expression in gastric cancer. Gut. (2019) 68:1764–73. doi: 10.1136/gutjnl-2018-316324. PMID: 30661053

[5] Shen D-D Pang J-R Bi Y-P Zhao L-F Li Y-R Zhao L-J . LSD1 deletion decreases exosomal PD-L1 and restores T-cell response in gastric cancer. Mol Cancer. (2022) 21:75. doi: 10.1186/s12943-022-01557-1. PMID: 35296335 PMC8925194

[6] Jia K Chen Y Sun Y Hu Y Jiao L Ma J . Multiplex immunohistochemistry defines the tumor immune microenvironment and immunotherapeutic outcome in CLDN18.2-positive gastric cancer. BMC Med. (2022) 20:223. doi: 10.1186/s12916-022-02421-1. PMID: 35811317 PMC9272556

[7] Wang Y Wang H Shi T Song X Zhang X Zhang Y . Immunotherapies targeting the oncogenic fusion gene CLDN18-ARHGAP in gastric cancer. EMBO Mol Med. (2024) 16:2170–87. doi: 10.1038/s44321-024-00120-3. PMID: 39164472 PMC11393071

[8] Cappell KM Kochenderfer JN . A comparison of chimeric antigen receptors containing CD28 versus 4-1BB costimulatory domains. Nat Rev Clin Oncol. (2021) 18:715–27. doi: 10.1038/s41571-021-00530-z. PMID: 34230645

[9] Wang Z Wang M Wang M Zhou R Deng X Ouyang X . From molecular design to clinical translation: dual-targeted CAR-T strategies in cancer immunotherapy. Int J Biol Sci. (2025) 21:2676. doi: 10.7150/ijbs.108036. PMID: 40303292 PMC12035882

[10] Hirabayashi K Du H Xu Y Shou P Zhou X Fucá G . Dual-targeting CAR-T cells with optimal co-stimulation and metabolic fitness enhance antitumor activity and prevent escape in solid tumors. Nat Cancer. (2021) 2:904–18. doi: 10.1038/s43018-021-00244-2. PMID: 34746799 PMC8570569

[11] Jiang H Shi Z Wang P Wang C Yang L Du G . Claudin18.2-specific chimeric antigen receptor engineered T cells for the treatment of gastric cancer. J Natl Cancer Inst. (2019) 111:409–18. doi: 10.1093/jnci/djy134. PMID: 30203099

[12] Nakayama I Qi C Chen Y Nakamura Y Shen L Shitara K . Claudin 18.2 as a novel therapeutic target. Nat Rev Clin Oncol. (2024) 21:354–69. doi: 10.1038/s41571-024-00874-2. PMID: 38503878

[13] Tsutsumi C Ohuchida K Yamada Y Shimada Y Imamura M Son K . Claudin18.2-positive gastric cancer-specific changes in neoadjuvant chemotherapy-driven immunosuppressive tumor microenvironment. Br J Cancer. (2025) 132(9):793–804. doi: 10.1038/s41416-025-02981-y. PMID: 40128286 PMC12041497

[14] Shitara K Lordick F Bang Y-J Enzinger P Ilson D Shah MA . Zolbetuximab plus mFOLFOX6 in patients with CLDN18.2-positive, HER2-negative, untreated, locally advanced unresectable or metastatic gastric or gastro-oesophageal junction adenocarcinoma (SPOTLIGHT): a multicentre, randomised, double-blind, phase 3 trial. Lancet. (2023) 401:1655–68. doi: 10.1016/s0140-6736(23)00620-7. PMID: 37068504

[15] Shah MA Shitara K Ajani JA Bang Y-J Enzinger P Ilson D . Zolbetuximab plus CAPOX in CLDN18.2-positive gastric or gastroesophageal junction adenocarcinoma: the randomized, phase 3 GLOW trial. Nat Med. (2023) 29:2133–41. doi: 10.1038/s41591-023-02465-7. PMID: 37524953 PMC10427418

[16] Zhao Z Condomines M van der Stegen SJ Perna F Kloss CC Gunset G . Structural design of engineered costimulation determines tumor rejection kinetics and persistence of CAR T cells. Cancer Cell. (2015) 28:415–28. doi: 10.1016/j.ccell.2015.09.004. PMID: 26461090 PMC5003056

[17] Kawalekar OU O’Connor RS Fraietta JA Guo L McGettigan SE Posey AD . Distinct signaling of coreceptors regulates specific metabolism pathways and impacts memory development in CAR T cells. Immunity. (2016) 44:380–90. doi: 10.1016/j.immuni.2016.01.021. PMID: 26885860 PMC13498396

[18] Long AH Haso WM Shern JF Wanhainen KM Murgai M Ingaramo M . 4-1BB costimulation ameliorates T cell exhaustion induced by tonic signaling of chimeric antigen receptors. Nat Med. (2015) 21:581–90. doi: 10.1038/nm.3838. PMID: 25939063 PMC4458184

[19] Liu G Rui W Zhao X Lin X . Enhancing CAR-T cell efficacy in solid tumors by targeting the tumor microenvironment. Cell Mol Immunol. (2021) 18:1085–95. doi: 10.1038/s41423-021-00655-2. PMID: 33785843 PMC8093220

[20] Locke FL Filosto S Chou J Vardhanabhuti S Perbost R Dreger P . Impact of tumor microenvironment on efficacy of anti-CD19 CAR T cell therapy or chemotherapy and transplant in large B cell lymphoma. Nat Med. (2024) 30:507–18. doi: 10.1038/s41591-023-02754-1. PMID: 38233586 PMC10878966

[21] Drent E Poels R Ruiter R van de Donk NW Zweegman S Yuan H . Combined CD28 and 4-1BB costimulation potentiates affinity-tuned chimeric antigen receptor–engineered T cells. Clin Cancer Res. (2019) 25:4014–25. doi: 10.1158/1078-0432.ccr-18-2559. PMID: 30979735 PMC7477921

[22] Zhao X Yang J Zhang X Lu X-A Xiong M Zhang J . Efficacy and safety of CD28- or 4-1BB-based CD19 CAR-T cells in B cell acute lymphoblastic leukemia. Mol Ther Oncolytics. (2020) 18:272–81. doi: 10.1016/j.omto.2020.06.016. PMID: 32728615 PMC7378699

[23] Cheng Z Wei R Ma Q Shi L He F Shi Z . *In vivo* expansion and antitumor activity of coinfused CD28- and 4-1BB-engineered CAR-T cells in patients with B cell leukemia. Mol Ther. (2018) 26:976–85. doi: 10.1016/j.ymthe.2018.01.022. PMID: 29503204 PMC6079368

[24] Cook MS King E Flaherty KR Siddika K Papa S Benjamin R . CAR-T cells containing CD28 versus 4-1BB co-stimulatory domains show distinct metabolic profiles in patients. Cell Rep. (2025) 44(7):115973. doi: 10.1016/j.celrep.2025.115973. PMID: 40650909

[25] Wang R Dang M Harada K Han G Wang F Pool Pizzi M . Single-cell dissection of intratumoral heterogeneity and lineage diversity in metastatic gastric adenocarcinoma. Nat Med. (2021) 27:141–51. doi: 10.1038/s41591-020-1125-8. PMID: 33398161 PMC8074162

[26] Murad JP Kozlowska AK Lee HJ Ramamurthy M Chang W-C Yazaki P . Effective targeting of TAG72+ peritoneal ovarian tumors via regional delivery of CAR-engineered T cells. Front Immunol. (2018) 9:2268. doi: 10.3389/fimmu.2018.02268. PMID: 30510550 PMC6254427

[27] Fernández de Larrea C Staehr M Lopez AV Ng KY Chen Y Godfrey WD . Defining an optimal dual-targeted CAR T-cell therapy approach simultaneously targeting BCMA and GPRC5D to prevent BCMA escape–driven relapse in multiple myeloma. Blood Cancer Discov. (2020) 1:146–54. doi: 10.1158/2643-3230.bcd-20-0020. PMID: 33089218 PMC7575057

[28] Yan T Zhu L Chen J . Current advances and challenges in CAR T-cell therapy for solid tumors: tumor-associated antigens and the tumor microenvironment. Exp Hematol Oncol. (2023) 12:14. doi: 10.1186/s40164-023-00373-7. PMID: 36707873 PMC9883880

[29] Leary A Tan D Ledermann J . Immune checkpoint inhibitors in ovarian cancer: where do we stand? Ther Adv Med Oncol. (2021) 13:17588359211039899. doi: 10.1177/17588359211039899. PMID: 34422119 PMC8377306

[30] Bajor M Graczyk-Jarzynka A Marhelava K Burdzinska A Muchowicz A Goral A . PD-L1 CAR effector cells induce self-amplifying cytotoxic effects against target cells. J Immunother Cancer. (2022) 10:e002500. doi: 10.1136/jitc-2021-002500. PMID: 35078921 PMC8796262

[31] Liu H Ma Y Yang C Xia S Pan Q Zhao H . Severe delayed pulmonary toxicity following PD‐L1–specific CAR‐T cell therapy for non‐small cell lung cancer. Clin Trans Immunol. (2020) 9:e1154. doi: 10.1002/cti2.1154 PMC7546952 33072320

[32] Qi C Liu C Gong J Liu D Wang X Zhang P . Claudin18.2-specific CAR T cells in gastrointestinal cancers: phase 1 trial final results. Nat Med. (2024) 30:2224–34. doi: 10.1038/s41591-024-03037-z. PMID: 38830992

[33] Qi C Liu C Peng Z Zhang Y Wei J Qiu W . Claudin-18 isoform 2-specific CAR T-cell therapy (SATRI-CEL) versus treatment of physician’s choice for previously treated advanced gastric or gastro-oesophageal junction cancer (CT041-ST-01): a randomised, open-label, phase 2 trial. Lancet. (2025) 405:2049–60. doi: 10.1016/s0140-6736(25)00860-8. PMID: 40460847

[34] Di Stasi A Tey S-K Dotti G Fujita Y Kennedy-Nasser A Martinez C . Inducible apoptosis as a safety switch for adoptive cell therapy. N Engl J Med. (2011) 365:1673–83. doi: 10.1056/nejmoa1106152. PMID: 22047558 PMC3236370

[35] Larson RC Maus MV . Recent advances and discoveries in the mechanisms and functions of CAR T cells. Nat Rev Cancer. (2021) 21:145–61. doi: 10.1038/s41568-020-00323-z. PMID: 33483715 PMC8353572

[36] Guedan S Luu M Ammar D Barbao P Bonini C Bousso P . Time 2evolve: predicting efficacy of engineered T-cells–how far is the bench from the bedside? J Immunother Cancer. (2022) 10:e003487. doi: 10.1136/jitc-2021-003487. PMID: 35577501 PMC9115015

[37] Rankin AW Pham-Danis C Novak AJ Danis E Fry TJ Kohler ME . Increased NFAT activity with dual CAR stimulation in CD19xCD22 CAR T-cells is associated with decreased exhaustion and improved survival. J Immunother Cancer. (2025) 13:e011971. doi: 10.1136/jitc-2025-011971. PMID: 41238217 PMC12625949

[38] Feucht J Sun J Eyquem J Ho Y-J Zhao Z Leibold J . Calibration of CAR activation potential directs alternative T cell fates and therapeutic potency. Nat Med. (2019) 25:82–8. doi: 10.1038/s41591-018-0290-5. PMID: 30559421 PMC6532069

[39] Majumdar S Echelibe H Bettini M Bettini ML . The impact of CD3ζ ITAM multiplicity and sequence on CAR T-cell survival and function. Front Immunol. (2025) 15:1509980. doi: 10.3389/fimmu.2024.1509980. PMID: 39885989 PMC11779709

[40] James JR . Tuning ITAM multiplicity on T cell receptors can control potency and selectivity to ligand density. Sci Signaling. (2018) 11:eaan1088. doi: 10.1126/scisignal.aan1088. PMID: 29789296 PMC6517276

[41] Schoutrop E Poiret T El-Serafi I Zhao Y He R Moter A . Tuned activation of MSLN-CAR T cells induces superior antitumor responses in ovarian cancer models. J Immunother Cancer. (2023) 11:e005691. doi: 10.1136/jitc-2022-005691. PMID: 36746513 PMC9906404

[42] Wang P Wang Y Zhao X Zheng R Zhang Y Meng R . Chimeric antigen receptor with novel intracellular modules improves antitumor performance of T cells. Signal Transduction Targeted Ther. (2025) 10:20. doi: 10.1038/s41392-024-02096-5. PMID: 39809749 PMC11733243

[43] Yap M Boeffard F Clave E Pallier A Danger R Giral M . Expansion of highly differentiated cytotoxic terminally differentiated effector memory CD8+ T cells in a subset of clinically stable kidney transplant recipients: a potential marker for late graft dysfunction. J Am Soc Nephrol. (2014) 25:1856–68. doi: 10.1681/asn.2013080848. PMID: 24652799 PMC4116064

[44] Martinez GJ Pereira RM Äijö T Kim EY Marangoni F Pipkin ME . The transcription factor NFAT promotes exhaustion of activated CD8+ T cells. Immunity. (2015) 42:265–78. doi: 10.1016/j.immuni.2015.01.006. PMID: 25680272 PMC4346317

