# Supplementary material for: Systematic optimization of PD-L1 and CLDN18.2 CAR-T designs identifies a bicistronic dual-target, double-CD3ζ architecture with enhanced antitumor activity in gastric cancer
Source: Front Immunol. 2026 Jun 2;17:1805471. doi: 10.3389/fimmu.2026.1805471 (PMC13270025; doi:10.3389/fimmu.2026.1805471)
Supplement: Supplementary file 1 [file DataSheet1.pdf]

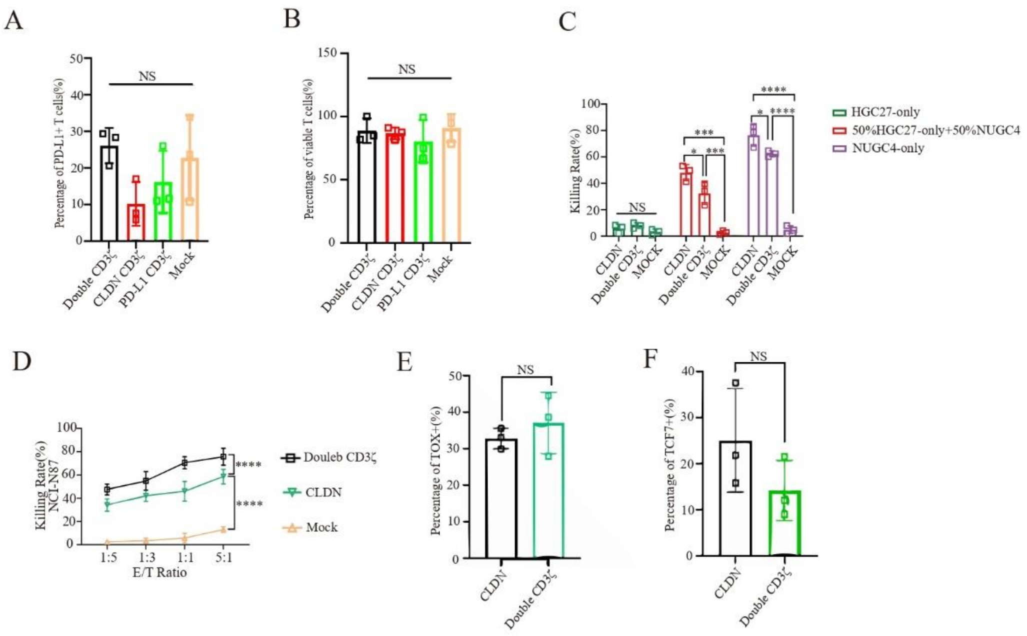

Supplementary Figure 1. Double CD3ζ CAR-T cells retain activity in heterogeneous antigen settings without evidence of increased PD-L1 expression, reduced viability, or altered TOX/TCF7 expression.

(A, B) PD-L1 expression and viability of CAR-T cells after 24 h stimulation. (C, D) Cytotoxicity after 24 h co-culture with NUGC4, HGC27, mixed (50% HGC27 + 50% NUGC4), and NCI-N87 target cells. (E, F) Intracellular TOX and TCF7 expression after four rounds of repeated antigen stimulation. Data are shown as mean ± SD. NS, not significant; *$P < 0.05$; ***$P < 0.001$; and ****$P < 0.0001$.
